# Self-Organization, Layered Structure, and Aggregation Enhance Persistence of a Synthetic Biofilm Consortium

**DOI:** 10.1371/journal.pone.0016791

**Published:** 2011-02-09

**Authors:** Katie Brenner, Frances H. Arnold

**Affiliations:** Division of Chemistry and Chemical Engineering, California Institute of Technology, Pasadena, California, United States of America; The University of Maryland, United States of America

## Abstract

Microbial consortia constitute a majority of the earth's biomass, but little is known about how these cooperating communities persist despite competition among community members. Theory suggests that non-random spatial structures contribute to the persistence of mixed communities; when particular structures form, they may provide associated community members with a growth advantage over unassociated members. If true, this has implications for the rise and persistence of multi-cellular organisms. However, this theory is difficult to study because we rarely observe initial instances of non-random physical structure in natural populations. Using two engineered strains of *Escherichia coli* that constitute a synthetic symbiotic microbial consortium, we fortuitously observed such spatial self-organization. This consortium forms a biofilm and, after several days, adopts a defined layered structure that is associated with two unexpected, measurable growth advantages. First, the consortium cannot successfully colonize a new, downstream environment until it self-organizes in the initial environment; in other words, the structure enhances the ability of the consortium to survive environmental disruptions. Second, when the layered structure forms in downstream environments the consortium accumulates significantly more biomass than it did in the initial environment; in other words, the structure enhances the global productivity of the consortium. We also observed that the layered structure only assembles in downstream environments that are colonized by aggregates from a previous, structured community. These results demonstrate roles for self-organization and aggregation in persistence of multi-cellular communities, and also illustrate a role for the techniques of synthetic biology in elucidating fundamental biological principles.

## Introduction

The vast majority of living biomass consists of single-celled organisms, but the existence of higher organisms demonstrates that interacting networks of cell populations can thrive despite competition between them [1], [2]. How nascent communities gain a growth advantage over unassociated individuals is an open question [2]–[9], but cell–cell interactions [10]–[14] and the formation of specific multi-cellular structures [15]–[18] are thought to contribute. Evaluating the role of physical structure in the initiation and persistence of natural consortia poses a causality dilemma [2], and *de novo* design of synthetic consortia that self-organize into specific structures is difficult. Thus, experimental studies of the formation and benefits of specific physical structures in mixed microbial communities are few.

We describe a synthetic symbiotic microbial consortium that allows us to address some of these questions. An advantage of using synthetic, or engineered, consortia for studies of this nature is that complex communal behaviors such as symbiosis can be implemented under defined and tunable experimental control [9], [19]–[21]. Although very simple relative to naturally-occurring microbial consortia, engineered ecosystems can nonetheless exhibit behaviors that mimic those found in nature and, because the interactions of engineered consortia can be controlled and more fully characterized, can provide insight into the development and persistence of natural communities.

It is useful to study the relationship between microbial community structure and persistence in biofilm communities for three primary reasons. First, biofilm spatial structure and productivity (total biomass accumulation) can be observed and quantified as a function of time using confocal laser scanning microscopy (CLSM) [22]. Second, stable micro-communities with very different properties and behaviors can form and persist within biofilms [23]–[26]. Finally, cells and sub-communities that detach from a biofilm subjected to fluid flow will flow downstream and may colonize downstream environments, where composition, spatial structure, and productivity can be observed. Thus, the effects of composition and spatial structure on the productivity of a consortium can be easily quantified when it grows as a biofilm. A structure that is beneficial should increase productivity in the local environment; it might also improve colonization or productivity when the consortium moves to downstream environments. The synthetic microbial consortium was engineered to rely on biofilm formation so that these effects could be measured.

## Results

**Figure 1:**
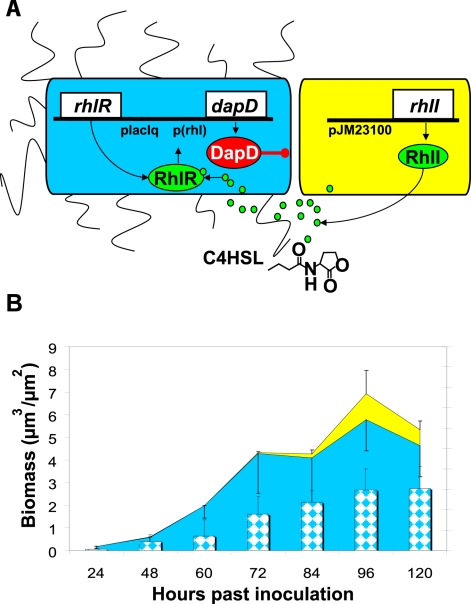
Design and initial characterization of the biofilm-forming consortium. (**A**) The synthetic symbiotic consortium. The blue population cannot synthesize diaminopimelate or lysine; when cultured without lysine or diaminopimelate, this population forms only a scant biofilm. The yellow population cannot form biofilms alone but is otherwise healthy. It synthesizes C4HSL, which diffuses freely and activates production of diaminopimelate in the blue population. Yellow cells become bound within the biofilm formed by the blue population and rescue growth. Together, the two populations form viable biofilms that persist. (**B**) The symbiotic consortium functions as designed. The blue population control forms a biofilm which eventually dies (blue bars), and the yellow population accumulates very little biomass (insignificant, not shown). When the yellow and blue populations are inoculated into a flow chamber in a 50/50 mixture, more biomass accumulates than in either control (solid yellow areas, total yellow biomass in the biofilm; solid blue areas, total blue biomass in the biofilm; the sum of blue and yellow areas is the overall total biomass in the biofilm at each time point; all errors are s.d.). All biofilm measurements were derived from images quantified with COMSTAT [22].

The synthetic symbiotic consortium consists of two engineered populations of *Escherichia coli* which are not viable alone, but can grow and form biofilms when grown together (Fig 1A). The first population, which is identified by constitutive expression of cyan fluorescent protein (CFP, the “blue population”), can form initially healthy biofilms. However, it cannot synthesize a set of metabolites critical for cell growth and division and therefore quickly dies. To engineer this strain we interrupted a critical pathway responsible for synthesis of diaminopimelate and lysine by deleting a key gene, *DapD* (*E. coli* MG1655Δ*DapD*) [27]. We then restored *DapD* under the control of a RhlR-dependent promoter. The transcription factor RhlR is activated by a small-molecule autoinducer, butanoyl-homoserine lactone (C4HSL) [28], which is provided by a second population. This “yellow population,” identified by constitutive expression of yellow fluorescent protein (YFP), cannot form biofilms alone but is otherwise healthy. To engineer this strain we identified genetic loci implicated in *E. coli* biofilm formation and, by trial-and-error, found a subset of these loci which when deleted significantly reduce biofilm formation without compromising growth rate. This set of deletions, removing genes involved in expression of type I pili, curli, colanic acid, and capsular polysaccharides, was concurrently identified elsewhere (*E. coli* MG1655Δ*fim,* Δ*wcaL–wza,* Δ*csgC-csgG)* [29]. Finally, strong constitutive production of RhlI, and thus of C4HSL, in this yellow population means that its presence up-regulates expression of DapD in biofilms formed by the blue population. Only when the yellow population becomes entangled within biofilms initially formed by the blue population can either population form enduring biofilms (Fig 1B). A 50/50 mixture of these two populations inoculated into biofilm flow chambers generates symbiotic biofilms that persist for at least 288 hours given a constant flow of fresh sterile nutrients; the timing of biomass growth and sloughing in these biofilms is repeatable over at least 120 hours (see Supporting Information S1 for more information regarding construction of the consortium and Supporting Information S2 for information regarding repeatability).

**Figure 2:**
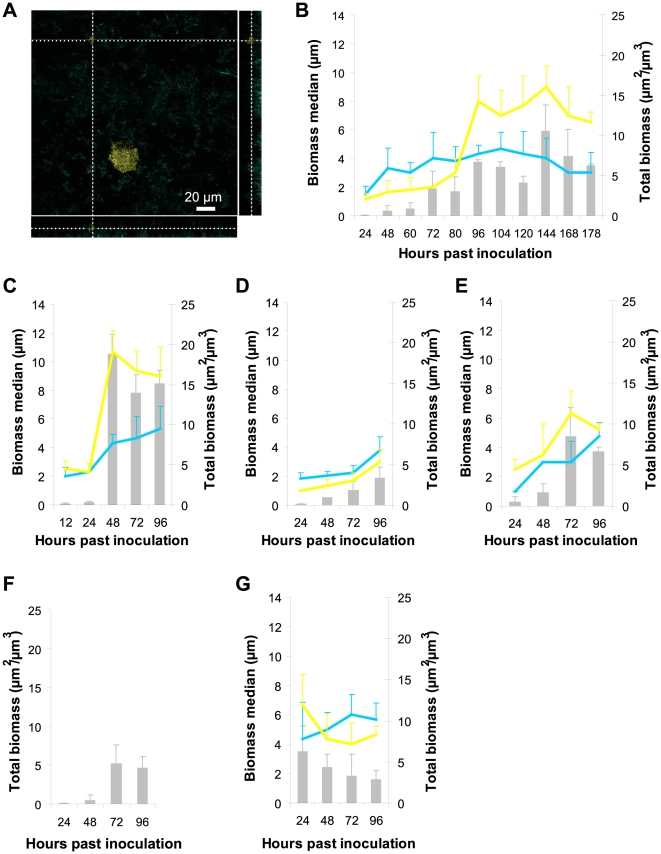
The consortium adopts a specific, layered structure which is associated with a growth advantage. (**A**) After 80 hours, the blue population remains primarily near the substrate while the yellow population forms clumps attached to the blue population, as shown in this cross-sectional projection taken at 1/3 the total height of the biofilm. (**B**) After 80 hours of growth, the yellow population begins to exhibit a consistently larger biomass median (yellow lines, throughout figure) than the blue population (blue lines, throughout figure), revealing that the yellow population grows further from the substrate while the blue population remains close to the substrate. (The biomass median indicates the average distance from the substrate at which cells of a given population are found.) Gray bars, plotted against the right-hand axis, indicate total biomass accumulation for the entire consortium at each time-point throughout the figure (errors throughout figure are s.d.). (**C**) Maximum total biomass accumulated by the downstream biofilm is double that in the initial biofilm (compare gray bars in [B] and [C]). Additionally, the downstream biofilm assumes the layered structure more quickly: the biomass medians reveal structure after 24 hours of growth. (**D**) When aggregates are disrupted prior to transfer, leaving all else constant, this treated effluent can form biofilms, but they never exhibit the layered structure or growth advantage. (**E**) The layered structure is recovered when the sorted blue-and-yellow aggregate fraction forms downstream biofilms. In fact, the consortium starts with this structure, exhibiting it by 24 hours after inoculation. Further, maximum total biomass accumulation is more than double the highest amount observed in the predecessor (illustrated in [D]), suggesting recovery of the growth advantage. (**F**) The single-cell fraction consists of more than 99% yellow cells, and thus neither the blue population nor the layered structure nor any growth advantage is evident in the downstream biofilm it forms. This biofilm accumulates less biomass than the biofilm formed by the aggregate fraction (compare to grey bars in [E]). (**G**) Here, effluent is taken from the treated biofilms, which are less productive and do not exhibit structure (illustrated in [D]). Although this effluent is left untreated, it forms initially dense, monomorphic, and primarily yellow downstream biofilms that do not exhibit layered structure. These biofilms consistently lose biomass.

After 80 hours of growth, the synthetic symbiotic biofilms always form a defined, layered physical structure. Yellow cells, initially randomly interspersed within a porous blue biofilm, grow away from the substrate to form clumps that are attached to the blue biomass. Pores previously present in the nascent, thin biofilm, which might have been filled by clonal expansion of either population, are filled entirely by blue cells which form a dense basement layer in the biofilm (Fig. 2A, B). The biomass medians—quantitative indicators of the locations of individual populations with respect to the substrate in mixed biofilms, as measured by CLSM—confirm this change (Fig. 2B). As this layered structure matures, the yellow clumps expand laterally to cover the blue biomass. Yellow cells are no longer found buried within the blue biomass; at steady state, the populations are vertically stratified, and the blue biomass forms a dense and uniform mat over the substrate, while the yellow biomass forms an uneven layer attached to this blue mat.

There is precedent for this type of self-organization in microbial consortia: after *Acinetobacter* and *Pseudomonas putida* are co-cultured in laboratory biofilms for several days, *P. putida* forms clumps on *Acinetobacter*. This structure provides a growth advantage for *P. putida* when carbon is scarce [30]. A growth advantage is associated with the structure of the synthetic symbiotic biofilm consortium as well. Samples of symbiotic biofilm effluent (containing the same numbers of viable cells) taken immediately before and after the layered structure emerges were transferred into fresh, downstream environments (experimental schematics can be found in Supporting Information S3). Effluent taken from the symbiotic consortium just prior to emergence of structure is unable to productively colonize a downstream environment. The resulting biofilm accumulates less biomass than the blue control monoculture biofilm shown in Fig. 2B. Effluent taken before the structure forms may contain primarily sessile or unhealthy cells. In contrast, effluent from the symbiotic consortium already exhibiting layered structure successfully colonizes a downstream flow chamber. This successful downstream biofilm recapitulates its predecessor's structure, but assumes the structure more quickly and accumulates more total biomass (Fig. 2C). In particular, the maximal total biomass in the successful downstream biofilm is always at least double the highest amount observed in the predecessor (Fig. 2B, C). Thus, the layered structure provides two clear growth advantages for this consortium. First, the structured consortium can colonize downstream environments, and second, when the structure forms in new environments the consortium accumulates more total biomass than it did in the initial environment. The most significant measurable benefits of this structure are observed in the downstream communities.

We find the layered structure and growth advantage in downstream biofilms only when aggregates containing blue and yellow cells are preserved during effluent transfer. When we treat the effluent to disrupt aggregates prior to transfer, leaving yellow and blue viable cell counts constant, this treated effluent can form biofilms, but they never exhibit the layered structure or growth advantage (Fig. 2D, Supporting Information S3). However, we can sort effluent from these unproductive biofilms and collect two fractions, aggregates containing both blue and yellow cells and single, unassociated cells (Supporting Information S3, information regarding sorting can be found in Supporting Information S4). The layered structure is recovered when the blue-and-yellow aggregate fraction forms biofilms downstream, and biomass accumulation is double the highest amount observed in the predecessor, suggesting recovery of the growth advantage (compare Figs. 2D, E). The single-cell fraction consists of more than 99% yellow cells, and thus neither the blue population nor structure nor any growth advantage is evident in the downstream biofilm it forms (Fig. 2F). Furthermore, although the blue-and-yellow aggregates are sorted from the effluent of unproductive biofilms, that same effluent—left untreated and unsorted—forms unusual downstream biofilms that are never otherwise observed (Supporting Information S3). These are initially dense, monomorphic, and primarily yellow. They do not exhibit defined structure and, after starting with significant amounts of biomass, these biofilms consistently lose biomass rather than accumulate it (Fig. 2G). We conclude that blue-and-yellow aggregates are necessary to preserve and convey the beneficial layered structure and growth advantage into downstream environments, but their abilities can be modulated or destroyed by other constituents in the effluent.

## Discussion

Biofilms can propagate when single cells dissociate from the outer regions of mature biofilms and adhere downstream, and this is considered the primary mode by which biofilms spread [31], [32]. Our results suggest this as one mechanism of proliferation. The regions of the symbiotic biofilm that are exposed to flow are predominantly populated by yellow cells, and more than 99% of cells in the single-cell fraction are yellow (Supporting Information S4). This fraction can form biofilms downstream, indicating that the yellow population adapts to adhere better, but these biofilms are weak and monomorphic (Fig. 2F). However, when effluent is never treated or sorted, the whole consortium colonizes downstream environments through multiple transfers and these biofilms accumulate more biomass than the initial biofilm. Therefore, aggregates that dissociate from upstream biofilms and colonize downstream environments enhance overall growth and proliferation of this consortium. These results indicate that a critical mechanism by which microbial communities propagate is the movement of aggregated members into downstream environments.

What are these aggregates, and how do they work? Aggregates preserve the physical relationship between the blue and yellow populations, and enhance yellow adhesion in downstream biofilms. Aggregates are distinct but conjoined clusters of blue and yellow biomass (Supporting Information S4, information regarding aggregate composition can be found in Supporting Information S5). When aggregates are transferred, average downstream biofilms contain five times more yellow biomass, after initial adhesion, than when aggregates are disrupted, even though both inocula contain the same numbers of blue and yellow cells (Supporting Information S3, S4, S5). Aggregates appear to be pre-organized pieces of the layered structure that quickly grow to recapitulate it in new environments. Additionally, it is possible that the proximity of blue and yellow cells in the aggregates enhances collaboration and therefore productivity (this is difficult to assess and remains untested).

We have used an engineered symbiotic microbial consortium to explore spatial self-organization and its benefits to a microbial community. Here, two symbiotic populations of *E. coli* grow to form a defined, layered structure which provides a growth advantage to both. This engineered consortium allowed us to observe the critical roles of self-organization, layering, and aggregation in the growth, movement, and ability of a microbial consortium to colonize new environments. The persistence assured by aggregates allows evolution and adaptation of interacting microbial communities despite environmental disruptions. It may eventually be possible to use engineered consortia like this one to determine how relationships between interacting, co-evolving populations are enhanced and preserved by particular physical structures.

## Materials and Methods

### Strains

To construct the knockout strains of *E. coli* MG1655, we used recombination with the lambda red recombinase plasmid pKD46, as outlined in [33]. To compromise metabolism we deleted *dapD* to make *E. coli* MG1655Δ*DapD* [27]. Biofilm formation was compromised by deleting the csgAB and csgDEFG operons and the Δ*wcaL–wza* gene locus in strain AAEC191 to create *E. coli* MG1655Δ*fim,* Δ*wcaL–wza,* Δ*csgC-csgG*. Further details are found in Supporting Information S1.

### Plasmids

The engineered plasmid in the blue population encodes constitutive production of RhlR from the *p(LacIq)* promoter with strong ribosome binding site RBSII, and RhlR-dependent expression of DapD from the qsc119 promoter with the weak ribosome binding site RBSH. DapD was expressed with an LVA degradation tag (DapD-LVA). The engineered plasmid in the yellow population encodes very strong constitutive expression of RhlI from the strong promoter p(JM2300) coupled with RBSII. More information is available in Supporting Information S1.

### Growth conditions

Throughout all experiments, cultures and biofilms were grown at 30°C in M9-AADO (Amino Acid Drop Out) medium without lysine, containing 50 µg ml^−1^ kanamycin and 20 µg ml^−1^ tetracycline to maintain the engineered and the marker plasmids, respectively [34].

#### M9-AADO (per liter)

200 mL 5xM9, 100 mL 10x AADO Solution without lysine, 2 mM MgSO_4_, 0.5% glycerol, 0.01% thymine.

#### 5x M9 (per liter)

18 g anhydrous Na_2_HPO_4_, 15g KH_2_PO_4_, 5g NH_4_Cl, 2.5g NaCl.

#### 10x Amino Acid Drop Out Solution (without Lysine, per liter)

300 mg l-isoleucine; 1500 mg l-valine; 200 mg l-adenine hemisulfate salt; 200 mg l-arginine HCl; 200 mg l-histidine HCl monohydrate; 1000 mg l-leucine; 200 mg l-methionine; 500 mg l-phenylalanine; 2000 mg l-threonine; 200 mg l-tryptophan; 300 mg l-tyrosine; 200 mg l-uracil.

### Biofilm Experiments

The biofilm flow apparatus was described in detail previously [35], and additional details are found in Supporting information S6. To begin initial biofilms, separate overnight cultures of blue and yellow populations were shaken, in M9-AADO medium with antibiotics as detailed above, to saturation. Cultures of the blue population were supplemented with 10 µM C4HSL (Sigma, O9945). Cultures were centrifuged at 4000 RPM for 8 minutes, cells were resuspended in 1 mL 0.9% NaCl solution containing the same antibiotics, then diluted into 0.9% NaCl solution with the antibiotics to an OD_600_ of 0.07, which corresponds to approximately 4×10^7^ cells mL^−1^. 1 mL of a 50/50 mixture of blue and yellow cells was inoculated into each flow lane for experimental replicates. Control biofilms were started with a 50/50 mixture of blue or yellow cells and 0.9% NaCl solution.

Prior to inoculation each 1×4×40 mm lane of each flow chamber (Stovall Life Sciences, ACFL0001) was incubated for at least 90 minutes at 37°C with 200 µL of a solution of 10 mg mL^−1^ bovine ribonuclease B (Sigma, R7884) suspended in 0.02 M bicarbonate buffer. Each lane was then quenched with 200 µL of 0.2% bovine serum albumin (Sigma, A4503). Flow of M9-AADO with antibiotics through the flow chambers was initiated for five minutes prior to inoculation. After inoculation, flow chambers were incubated glass-coverslip-down for 4 minutes, and then flow was reinstated for 4 minutes prior to returning the flow chambers to the upright position. The flow rate of medium through each lane was approximately 230 µL min^−1^, and flow cells were incubated at 30°C±2°C throughout the length of each experiment. Medium reservoirs were replaced every 12 hours to ensure freshness of the antibiotics.

### Treated and Untreated Biofilms

To begin both treated and untreated downstream biofilms, effluents from three separate replicates (in separate lanes) of the type of biofilm to be propagated were mixed. This mixture was divided into treated and untreated cases. In the untreated case, OD_600_ was adjusted to 0.07 as necessary, and 1 mL was inoculated into each fresh flow lane. In the treated case, the effluent was vortexed for 5 minutes and then passed through a 40 µm cell strainer (BD Falcon, 352340) before the effluent was adjusted to 0.07 and 1 mL was inoculated into each fresh flow lane. The treated and untreated effluents were also plated in parallel with inoculation to confirm that they contained equal numbers of blue and yellow cells.

### Imaging and image processing

Images of the biofilms were captured with a Zeiss 510 upright confocal laser scanning microscope (CLSM), controlled by Carl Zeiss AIM. A Zeiss Achroplan 40×/0.8 W objective was used to capture all images, images were captured with 512×512 pixel resolution, and all image stacks were captured with identical pinhole and gain settings. eCFP excitation: 458 nm Argon laser, emission filter: BP 480–520 nm. eYFP excitation: 514 nm Argon laser, emission filter: LP 530 nm.

All image-based measurements were calculated using the COMSTAT biofilm image processing package in Matlab [22]. At least three biological replicates were grown at a time for each condition, and every condition was repeated on at least two different days. Averages were taken of COMSTAT results from at least three randomly selected images, taken at a variety of locations within the flow lane. Additions to the COMSTAT software enabled calculations of biomass median, as detailed in Supporting Information S7 [22]. The biomass median measures the average distance from the substrate at which cells of a given population are found; if populations are well-mixed the medians will be the same, and if they are stratified one median will be significantly larger than the other.

## Supporting Information

Supporting Information S1 Construction of the synthetic biofilm-forming consortium.
(DOC)

Supporting Information S2 Repeatability and stability of the engineered symbiotic biofilm.
(DOC)

Supporting Information S3 Experimental schematics.
(DOC)

Supporting Information S4 FACS was used to separate aggregates and single cells.
(DOC)

Supporting Information S5 Aggregates are clusters containing blue and yellow biomass.
(DOC)

Supporting Information S6 Biofilm preparation, inoculation, and treatment procedures.
(DOC)

Supporting Information S7 COMSTAT calculation of biomass median.
(DOC)
