# Supplementary material for: Self-Organization, Layered Structure, and Aggregation Enhance Persistence of a Synthetic Biofilm Consortium
Source: PLoS One. 2011 Feb 9;6(2):e16791. doi: 10.1371/journal.pone.0016791 (PMC3036657; doi:10.1371/journal.pone.0016791)
Supplement: Supporting Information S1 — Construction of the synthetic biofilm-forming consortium. (DOC) [file pone.0016791.s001.doc]

Self-Organization, Layered Structure, and Aggregation Enhance Persistence of a Synthetic Biofilm Consortium

**Supporting Information S1:**

**Construction of the synthetic biofilm-forming consortium**

**S1.1 Constructing the metabolically-compromised (blue) strain**

The symbiotic biofilm consortium consists of two engineered populations of *Escherichia coli* MG1655, one of which is deficient in biofilm formation but otherwise healthy, while the other is metabolically compromised but capable of biofilm formation (Fig. 1A). As mentioned in Materials and Methods, to construct the “blue” strain, we first deleted *dapD*, the gene encoding tetrahydrodipicolinate N-succinyltransferase, to create strain MG1655*∆DapD* (36). We used the lambda red recombinase plasmid pKD46 and the procedure outlined in (37). The chromosomal inserts to replace *dapD* were constructed by PCR with template plasmid pKD4. The primers used were:

dapD-P1-fwd: 5’-ATGCAGCAGTTACAGAACATTATTGAAACCGCTTTTGAACGCCGGTGTAGGCTGGAGCTGCTTC

and dapD-P2-rev: 5’-TTAGTCGATGGTACGCAGCAGTTCGTTAATGCCGACTTTGCCGCATATGAATATCCTCCTTA;

Recombinant clones were selected with 50 μg ml^-1^ kanamycin, cured at 42° C, and tested with colony PCR reactions using internal primers to confirm the presence of the kanamycin resistance gene and absence of the target genes. Plasmid pCP20, containing the Flp recombinase, was transformed into cells containing successful kanamycin inserts to remove the inserts (38). Finally, clones were again cured at 42° C to remove pCP20, and the same colony PCR reactions with internal primers were repeated to confirm the deletions of target genes and of the kanamycin resistance gene insert.

**S1.2 Constructing the biofilm-deficient (yellow) strain**

To construct a biofilm-deficient version of MG1655, we deleted three groups of genes that are implicated in biofilm formation. We used the lambda red recombinase system detailed in S1.1 to make these deletions.

First, a factor which is involved in strong surface adhesion of *E. coli* biofilms, although not as clearly involved in the formation of three-dimensional structure, is the presence of type I pili (also called fimbriae). These cell-surface appendages form catch bonds whose binding is characteristically tighter under higher stress (39, 40). Genes responsible for fimbriae lie in the *fimA–fimH* locus, and the key gene whose product mediates catch bond formation is *fimH*. We used a mutant, *E. coli* AAEC191 (MG1655*∆fim*), lacking the entire locus for the purposes of this study (39). Many experimental studies use mannose-BSA to provide for catch-bond formation. Here, we used bovine ribonuclease B quenched with bovine serum albumin (BSA). In single mutants lacking the *fim* locus, we observed significantly less initial adhesion than in any other single mutant that we made.

Second, an important determinant of both initial adhesion and three-dimensional structure formation in *E. coli* biofilms is the presence of the cell-surface appendage called curli. Curli are important for initial adhesion to abiotic surfaces (41) and also for cell-cell adhesion that leads to three-dimensional structure formation (42, 43). In *E. coli*, curli are optimally expressed at 30ºC under low nutrient and low osmolarity conditions (44, 45), which are similar to the conditions we use in our study. When *csgA* and *csgD* were deleted from *E. coli* in previous studies that used similar conditions to our study, a sparse monolayer was the best biofilm formed by the resultant strain (43). Two operons, csgDEFG and csgAB, are responsible for the biosynthesis of curli monomers (CsgA) and their export. We deleted both operons entirely from strain AAEC191 to create strain *E. coli* MG1655*∆fim, ∆csgC-csgG*. Primers used were:

csgG-P1-fwd: 5’-TCAGGATTCCGGTGGAACCGACATATGGCGGTATTTCACCAGAATGTCATGTGTAGGCTGGAGCTGCTTC

and csgC-P2-rev: 5’-TTAAGACTTTTCTGAAGAGGGCGGCCATTGTTGTGATAAATGAAGTGACTGCATATGAATATCCTCCTTA;

A third factor, implicated in three-dimensional structure formation of *E. coli* biofilms but not in initial adhesion, is the presence of colanic acid (CA) (43). CA is an excreted polysaccharide that surrounds cells in biofilms and creates space between them which presumably allows for diffusion of nutrients and wastes, for communication between cells, and for growth. CA is a constituent of the “slime” that is commonly mentioned in macroscopic observations of biofilms. It is not clear whether *E. coli* MG1655 produces CA when it is sessile (43), but CA is important in determining the initial three-dimensional structure of MG1655 biofilms while cells are actively growing and dividing (43, 46). 19 genes in the *wca* locus are responsible for CA production and secretion. Genes encoding CA are most highly expressed at temperatures below 25ºC and in minimal medium with an accessible carbon source (47). These conditions are approximately those in our study so we deleted the entire locus *wcaL–wza* to create strain *E. coli* MG1655*∆fim, ∆wcaL–wza, ∆csgC-csgG.* Primers used were:

wza-P1-fwd: 5’-ATGATGAAATCCAAAATGAAATTGATGCCATTATTGGTGTCAGTAACCTTGTGTAGGCTGGAGCTGCTTC

and wcaL-P2-rev: 5’-CTATAAAGCCTGCAGCAAGCTGGCGAGTTCTCGATTGATCACCTGCTGGTCATATGAATATCCTCCTTA

**S1.3 Constructing the engineered plasmid in the blue strain**

The engineered plasmid in the blue population was constructed from pFNK202 which encodes constitutive expression of *RhlR* and RhlR-dependent expression of GFP (48). We initially simply replaced the *GFP* gene with the *dapD* gene. Proper function of the symbiotic consortium requires that very little DapD be present in blue cells in the absence of the yellow population, while the presence of yellow cells should restore biological levels of DapD to blue cells. However, minor expression (promoter “leakage”) of DapD allows the blue population to begin forming a sparse biofilm without C4HSL so that the symbiotic consortium can gain a foothold in the environment. We obtained an adequate basal expression level of *dapD*, with wild-type levels of biofilm formation in the presence of 10 µM C4HSL, by placing *dapD* under control of the RhlR-activated promoter p(qsc119) with ribosome binding site (RBS) H, and attaching an LVA degradation tag to DapD. We tried various other permutations of this arrangement, including a variety of RBS strengths, and expressing *dapD* with and without the LVA tag, but the combination we chose yielded optimal behavior. The additional presence of plasmid pMP4641 in this strain confers constitutive expression of eCFP (49).

**S1.4 Constructing the engineered plasmid in the yellow strain**

The yellow population contains an engineered plasmid encoding strong constitutive expression of the C4HSL synthase, RhlI. This engineered plasmid was constructed from pFNK102, which encodes constitutive expression of RhlI (48). Yellow cells must synthesize enough C4HSL to activate RhlR, and thereby to upregulate *dapD* expression, in the blue population early in the lifespan of the consortium so that the blue population does not die. The strong constitutive promoter p(lacIq), even coupled with the strong ribosome binding site RBSII, did not provide adequate C4HSL production to enable optimal function of the symbiotic consortium. We therefore replaced p(lacIq) with the stronger constitutive promoter pJ23100, and combined this with RBSII, to yield enough C4HSL in the biofilm environment to activate the symbiotic function. This strain also contains plasmid pMP4658, yielding constitutive expression of eYFP (49).

References

36. Richaud C, Richaud F, Martin C, Haziza C, Patte JC (1984) Regulation of expression and nucleotide sequence of the escherichia coli dapd gene. *J Biol Chem* **259,** 14824-8.

37. Datsenko KA, Wanner BL (2000) One-step inactivation of chromosomal genes in escherichia coli k-12 using pcr products. *Proc Natl Acad Sci U S A* **97,** 6640-5.

38. Cherepanov PP, Wackernagel W (1995) Gene disruption in escherichia coli: Tcr and kmr cassettes with the option of flp-catalyzed excision of the antibiotic-resistance determinant. *Gene* **158,** 9-14.

39. Blomfield IC, McClain MS, Eisenstein BI (1991) Type 1 fimbriae mutants of escherichia coli k12: Characterization of recognized afimbriate strains and construction of new fim deletion mutants. *Mol Microbiol* **5,** 1439-45.

40. Thomas W, et al. (2006) Catch-bond model derived from allostery explains force-activated bacterial adhesion. *Biophys J* **90,** 753-64.

41. Prigent-Combaret C, et al. (2001) Complex regulatory network controls initial adhesion and biofilm formation in escherichia coli via regulation of the csgd gene. *J Bacteriol* **183,** 7213-23.

42. Vidal O, et al. (1998) Isolation of an escherichia coli k-12 mutant strain able to form biofilms on inert surfaces: Involvement of a new ompr allele that increases curli expression. *J Bacteriol* **180,** 2442-9.

43. Prigent-Combaret C, et al. (2000) Developmental pathway for biofilm formation in curli-producing escherichia coli strains: Role of flagella, curli and colanic acid. *Environ Microbiol* **2,** 450-64.

44. Olsen A, Jonsson A, Normark S (1989) Fibronectin binding mediated by a novel class of surface organelles on escherichia coli. *Nature* **338,** 652-5.

45. Olsen A, Arnqvist A, Hammar M, Sukupolvi S, Normark S (1993) The rpos sigma factor relieves h-ns-mediated transcriptional repression of csga, the subunit gene of fibronectin-binding curli in escherichia coli. *Mol Microbiol* **7,** 523-36.

46. Xavier JB, Foster KR (2007) Cooperation and conflict in microbial biofilms. *Proc Natl Acad Sci U S A* **104,** 876-81.

47. Markovitz A (1977) in *Surface carbohydrates of the prokaryotic cell*, ed. Sutherland IW (Academic Press, New York), Vol. 1, pp. 415-462.

48. Brenner K, Karig DK, Weiss R, Arnold FH (2007) Engineered bidirectional communication mediates a consensus in a microbial biofilm consortium. *Proc Natl Acad Sci U S A* **104,** 17300-4.

49. Bloemberg GV, Wijfjes AH, Lamers GE, Stuurman N, Lugtenberg BJ (2000) Simultaneous imaging of pseudomonas fluorescens wcs365 populations expressing three different autofluorescent proteins in the rhizosphere: New perspectives for studying microbial communities. *Mol Plant Microbe Interact* **13,** 1170-6.
