# Supplementary material for: Self-Organization, Layered Structure, and Aggregation Enhance Persistence of a Synthetic Biofilm Consortium
Source: PLoS One. 2011 Feb 9;6(2):e16791. doi: 10.1371/journal.pone.0016791 (PMC3036657; doi:10.1371/journal.pone.0016791)
Supplement: Supporting Information S2 — Repeatability and stability of the engineered symbiotic biofilm. (DOC) [file pone.0016791.s002.doc]

Self-Organization, Layered Structure, and Aggregation Enhance Persistence of a Synthetic Biofilm Consortium

**Supporting Information S2:**

Repeatability and stability of the engineered symbiotic biofilm

The blue and yellow populations coexist for periods of up to 288 hours in the biofilm environment (experiments were terminated after 288 hours; images were not collected past 178 hours). Biofilm sloughing and growth phases occur at repeatable times from experiment to experiment, even when experiments are conducted months apart (Fig A, below). Repeatability over the first 120 hour time period—the length of time for which we obtained the most independent replicates—was confirmed with analysis of variance (ANOVA), F0.05(3,28) = 0.47 < Fcrit = 2.947, P = 0.70. This demonstrates that behavior of the engineered consortium is repeatable over at least 120 hours, which is longer than necessary to validate all quantitative comparisons that we make. However, some weeks all biofilms accumulated slightly more total biomass than other weeks (see November versus December in Figure B1), probably due to changes in incubation temperature, which was difficult to precisely control. The average relationships between biofilms that we compare in this work were repeatable from week to week even when total biomass accumulation was not equivalent.

**Figure A** Mean total biomass in µm, with respect to hours past inoculation, for independent symbiotic biofilms cultured months apart (Nov, November; Dec, December; JanI, early January; JanII, late January). These data reveal that biomass accumulation, including timing of growth and dispersal phases, of the symbiotic ecosystem is repeatable between independent biological replicates (P = 0.7).
