# Supplementary material for: Self-Organization, Layered Structure, and Aggregation Enhance Persistence of a Synthetic Biofilm Consortium
Source: PLoS One. 2011 Feb 9;6(2):e16791. doi: 10.1371/journal.pone.0016791 (PMC3036657; doi:10.1371/journal.pone.0016791)
Supplement: Supporting Information S3 — Experimental schematics. (DOC) [file pone.0016791.s003.doc]

Self-Organization, Layered Structure, and Aggregation Enhance Persistence of a Synthetic Biofilm Consortium

**Supporting Information S3:**

Experimental schematics

**A**

B

**C**

**Figure S3** Experimental schematics. (**A**) Downstream biofilms grow and exhibit a growth advantage only if the predecessor has already established layered structure. (**B**) Treatment to remove aggregates, leaving all else constant, prevents formation of structure and growth advantage in downstream biofilms. (**C**) Cell sorting demonstrates that the aggregates are necessary and sufficient to seed biofilms that exhibit structure and growth advantage.
