# Supplementary material for: Self-Organization, Layered Structure, and Aggregation Enhance Persistence of a Synthetic Biofilm Consortium
Source: PLoS One. 2011 Feb 9;6(2):e16791. doi: 10.1371/journal.pone.0016791 (PMC3036657; doi:10.1371/journal.pone.0016791)
Supplement: Supporting Information S4 — FACS was used to separate aggregates and single cells. (DOC) [file pone.0016791.s004.doc]

Self-Organization, Layered Structure, and Aggregation Enhance Persistence of a Synthetic Biofilm Consortium

**Supporting Information S4:**

FACS was used to separate aggregates and single cells


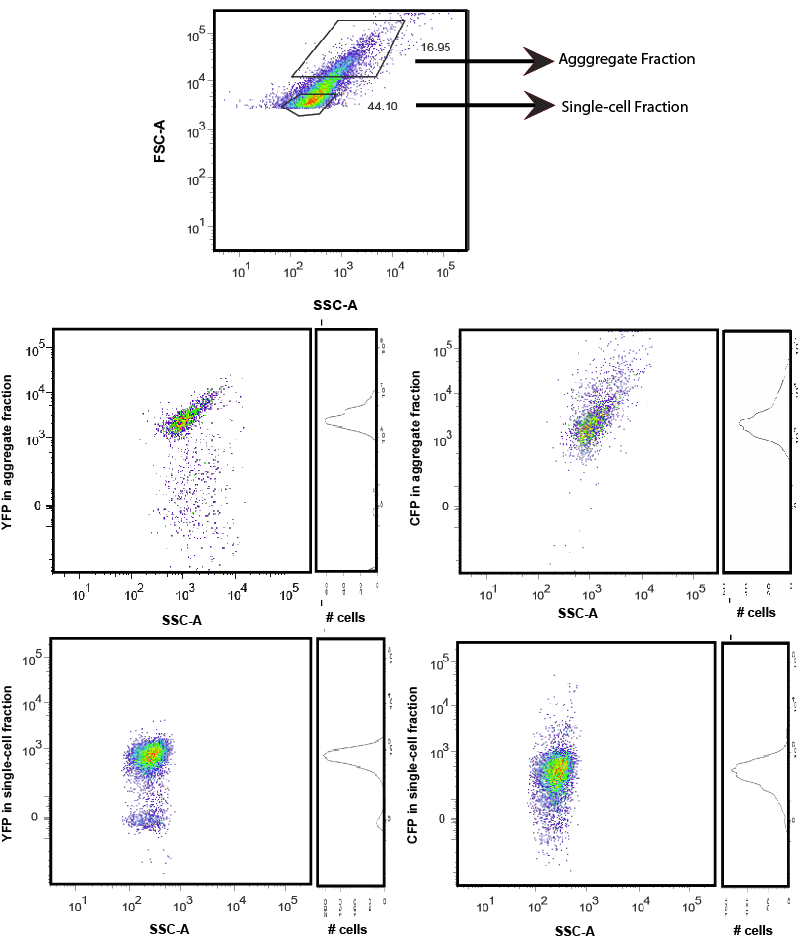


**Supporting Figure S4** FACS data. The single-cell fraction is conservatively gated to contain only single cells (based upon fluorescent and no-stain controls). This fraction fluoresces in the yellow range, with fewer than one percent of events occurring in the blue range, and a subpopulation that appears to lack fluorescence entirely (this could be dead cells—they do not appear to adhere). The aggregate fraction contains groups of cells of unidentified shape. These were gated to fluoresce in the yellow and blue ranges, and a great majority of these aggregates certainly contain both blue and yellow biomass.
