# Supplementary material for: Self-Organization, Layered Structure, and Aggregation Enhance Persistence of a Synthetic Biofilm Consortium
Source: PLoS One. 2011 Feb 9;6(2):e16791. doi: 10.1371/journal.pone.0016791 (PMC3036657; doi:10.1371/journal.pone.0016791)
Supplement: Supporting Information S5 — Aggregates are clusters containing blue and yellow biomass. (DOC) [file pone.0016791.s005.doc]

Self-Organization, Layered Structure, and Aggregation Enhance Persistence of a Synthetic Biofilm Consortium

**Supporting Information S5:**

**Aggregates are clusters containing blue and yellow biomass**


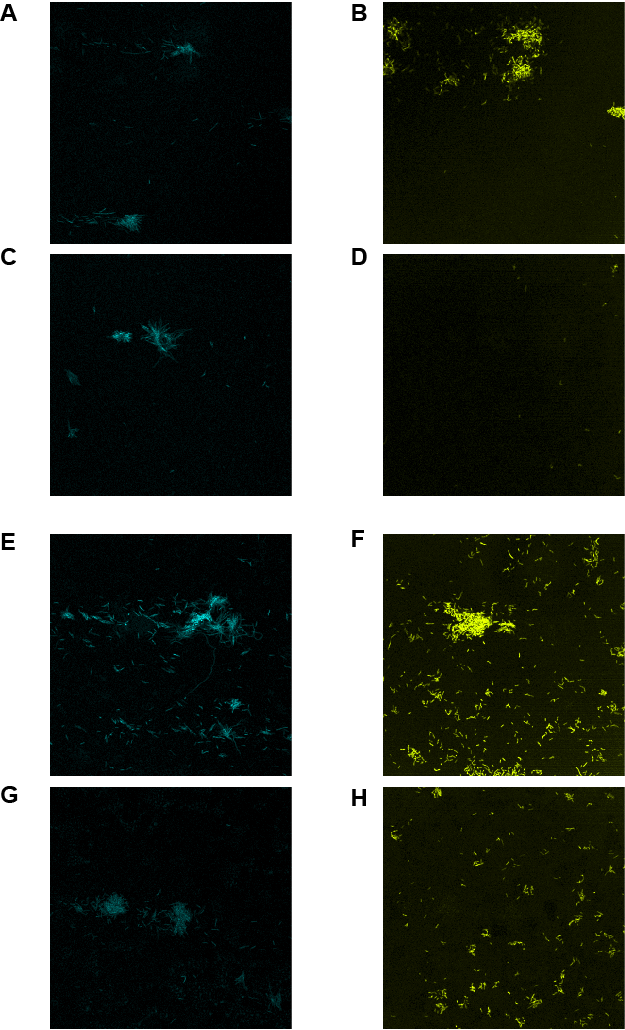


**Supporting Figure S5** Blue and yellow component images of aggregates adhering to the substrate. (**A**) Blue and (**B**) yellow biomass is co-localized at the substrate after inoculation in downstream biofilms formed by untreated effluent. In contrast, in treated-effluent biofilms (**C**) blue biomass is still clustered but (**D**) only a few yellow cells are present, distributed across the substrate, and not associated with blue cells. After 24 hours of growth, (**E**) blue and (**F**) yellow continue to be co-localized and to grow well in untreated biofilms. In contrast, (**G**) blue and (**H**) yellow have grown less and are less healthy in the treated case after 24 hours.
