# Supplementary material for: Self-Organization, Layered Structure, and Aggregation Enhance Persistence of a Synthetic Biofilm Consortium
Source: PLoS One. 2011 Feb 9;6(2):e16791. doi: 10.1371/journal.pone.0016791 (PMC3036657; doi:10.1371/journal.pone.0016791)
Supplement: Supporting Information S6 — Biofilm preparation, inoculation, and treatment procedures. (DOC) [file pone.0016791.s006.doc]

Self-Organization, Layered Structure, and Aggregation Enhance Persistence of a Synthetic Biofilm Consortium

**Supporting Information S6:**

**Biofilm preparation, inoculation, and treatment procedures**

The biofilm flow apparatus was described previously in (48) with two exceptions. First, here inoculation was performed into the Tygon tubing via inoculation ports installed into three-way connectors one inch upstream of each flow lane (connectors, Cole Parmer, A-06365-88; inoculation ports, Fisher Scientific, 03-215-5). Second, two inches of tubing upstream of flow lanes, including the inoculation port, was removed within 48 hours of inoculation to prevent upstream biofilm formation from affecting results within the flow cells.
