# Supplementary material for: Self-Organization, Layered Structure, and Aggregation Enhance Persistence of a Synthetic Biofilm Consortium
Source: PLoS One. 2011 Feb 9;6(2):e16791. doi: 10.1371/journal.pone.0016791 (PMC3036657; doi:10.1371/journal.pone.0016791)
Supplement: Supporting Information S7 — COMSTAT calculation of biomass median. (DOC) [file pone.0016791.s007.doc]

Self-Organization, Layered Structure, and Aggregation Enhance Persistence of a Synthetic Biofilm Consortium

**Supporting Information S7:**

**COMSTAT calculation of biomass median**

Changes made to COMSTAT to calculate the biomass median are in the following excerpt of Matlab code:

function [y,bmp,hs]=biomass_func(bb,xyarea,voxel)

count=0; hs=0; layersum=0; bmpcount=0; halfsum=0;

for side=1:size(bb,3)

loc1=bb(:,:,side)>0;

count=count+sum(sum(loc1));

end

y=count*voxel/(xyarea*size(bb,1)*size(bb,2));

halfcount=count/2;

for side=1:size(bb,3)

loc1=bb(:,:,side)>0;

layersum=sum(sum(loc1));

bmp(side)=(layersum/count)*100;

end

for side=1:size(bb,3)

loc1=bb(:,:,side)>0;

halfsum=halfsum+sum(sum(loc1));

if halfsum>=halfcount

hs=side;

break;

end

end
